# Genetic diversity of a successful colonizer: isolated populations of *Metrioptera roeselii* regain variation at an unusually rapid rate

**DOI:** 10.1002/ece3.1005

**Published:** 2014-03-07

**Authors:** Peter Kaňuch, Åsa Berggren, Anna Cassel-Lundhagen

**Affiliations:** 1 Department of Ecology, Swedish University of Agricultural SciencesBox 7044, 75007, Uppsala, Sweden; 2 Institute of Forest Ecology, Slovak Academy of SciencesĽ. Štúra 2, 96053, Zvolen, Slovakia

**Keywords:** Bottleneck, founder effect, gene flow, invasiveness, Orthoptera, temporal change

## Abstract

Newly founded isolated populations need to overcome detrimental effects of low genetic diversity. The establishment success of a population may therefore depend on various mechanisms such as assortative mating, purging of deleterious alleles, creation of new mutations and/or repeated inflow of new genotypes to reduce the effects of inbreeding and further loss of genetic variation. We compared the level of genetic variation in introduced populations of an insect species (*Metrioptera roeselii*) far beyond its natural distribution with levels found in their respective founder populations and coupled the data with timing since establishment. This allowed us to analyze if the introduced populations showed signs of temporal changes in genetic variation and have made it possible to evaluate underlying mechanisms. For this, we used neutral genetic markers, seven microsatellite loci and a 676–bp-long sequence of the mtDNA COI gene. All tested indices (allelic richness, unbiased expected heterozygosity, effective size, haplotype diversity, and nucleotide diversity) except inbreeding coefficient had significantly higher values in populations within the founding populations inside the continuous area of the species distribution compared with the introduced populations. A logarithmic model showed a significant correlation of both allelic richness and unbiased expected heterozygosity with age of the isolated populations. Considering the species' inferred colonization history and likely introduction pathways, we suggest that multiple introductions are the main mechanism behind the temporal pattern observed. However, we argue that influences of assortative mating, directional selection, and effects of an exceptional high intrapopulation mutation rate may have impacts. The ability to regain genetic diversity at this level may be one of the main reasons why *M. roeselii* successfully continue to colonize northern Europe.

## Introduction

With increasing human activities, particularly in agriculture, trade, and transport, the numbers of introductions of non-native species have increased dramatically during the second half of the 20th century (di Castri 1989). If introduced species are competitive and/or lack natural enemies, they may become invasive and result in major detrimental effects on native biodiversity (see reviews by Cox 2004; Whitney and Gabler 2008; Gren et al. 2009; Kenis et al. 2009). However, only 2–3% of introduced species become invasive after a successful colonization and naturalization in the local ecosystem (di Castri 1989). One reason for this low success rate may be that populations which are established from small numbers of individuals tend to lose genetic variation (Lande 1988; Lee 2002; Simberloff 2009). Increased effects of genetic drift and inbreeding in small populations tend to decrease heterozygosity and cause deleterious effects on individuals' fitness (inbreeding depression) (Lande 1988; Hedrick and Kalinowski 2000; Keller and Waller 2002). Inbreeding depression can, in addition, result in more pronounced losses of genetic variation over time causing a so-called ‘extinction vortex’ (Tanaka 2000).

A major question in invasion biology is how newly founded and subsequently isolated populations overcome the detrimental effects of low genetic diversity, the so-called ‘genetic paradox’ (Allendorf and Lundquist 2003; Roman and Darling 2007). One argument has been that successfully colonizing species are introduced in large numbers or multiple times during a long time period causing no loss of genetic diversity in the founded population (Roman 2006; Dlugosch and Parker 2008). It is also possible that genetic variation is maintained via hybridization of several founding populations (Facon et al. 2008; Demandt 2010). Le Corre and Kremer (1998) demonstrated theoretically how the colonization process and its genetic consequences depend on the number of colonists and migrants. Their theoretical model suggests a gradual increase in genetic diversity (heterozygosity level) in established populations. The demographic increase is suggested to be most apparent in recent establishments where the initial phase often is characterized by species expansion in an unrestricted environment. Dlugosch and Parker (2008), on the other hand, found from empirical data that genetic diversity in isolated populations showed a U-shaped pattern, with a period of diversity decline followed by an increase to values similar to the source populations. The return of allelic richness in the populations to similar levels as their founder populations occurred in their study after approximately 150 generations. This pattern was explained by multiple introductions but, as isolated populations often have small effective sizes where effects of genetic drift are strong, the U-shaped pattern is plausible only if strong selection opposes genetic drift (O'Hara 2005; Dlugosch and Parker 2008).

Some species that establish successfully in new areas appear to not suffer despite that they have reduced genetic variation in their populations after the founder event (Dlugosch and Parker 2008). These colonizers apparently perform well with limited genetic variation also under regimes of permanently restricted gene flow (Lande 1988; Simberloff 2009). The capacity to adapt to new environments can depend on the species' ability to respond to natural selection, which in turn is determined by the genetic architecture of the founder population(s) (Lee 2002). Some species, which possess so-called ‘general-purpose genotypes’, characteristically thrive in a wide range of environmental conditions due to a high phenotypic plasticity (Parker et al. 2003; Chen et al. 2006).

When an isolated population originates from unintentional transport beyond the species range and subsequent immigration of new propagules (multiple introductions) is not possible, intrapopulation mutations are the only mechanism that can increase genetic diversity (Pannell and Charlesworth 2000). However, such processes are in general very slow, for example, the mutation rate per nucleotide site is on average 10^−8^ bp per generation in invertebrates (Lynch 2010). If new mutations are to be a significant contributor to genetic diversity, an exceptionally high mutation rate needs to be present. Interestingly, mutation rates do vary across organisms (Lynch 2010; Chapuis et al. 2012). It has been found that the rate of molecular evolution within a species group can depend on ecological factors such as variations in life-history and behavior, or on environmental factors such as range of distribution (Bromham 2011). As an example, a life in poor environmental conditions may select for high mutation rate as individuals can not invest heavily in DNA repair (Agrawal and Wang 2008). Such condition-dependent mutation rates can have large effects on population survival and performance.

In summary, the establishment success of a newly founded, presumably isolated, population may be due to recurrent colonization, accumulation of new mutations, and/or a high level of intrinsic phenotypic plasticity. In order to determine the relationship between temporal variation in a species' genetic diversity (i.e., loss or gain of genetic variation) and establishment success, knowledge about founder history, colonization pathways, and propagule pressure of species is needed (Le Corre and Kremer 1998; Lee 2002; Simmons and Thomas 2004; Chen et al. 2006). This is often challenging as this requires detailed knowledge about the focal species. This type of data is available for our study species Roesel's bush cricket, *Metrioptera roeselii* (Orthoptera: Tettigoniidae) from long-term and detailed studies. The species has extended its natural range by several long-distance introduction events far beyond the main distribution range during the last 130 years. This has resulted in new populations of *M. roeselii* being recorded in Sweden, Denmark, and England (Albrecht 1963; Ahlén 1995; Bavnhøj 1996; Nielsen 2000; Simmons and Thomas 2004; Gardiner 2009; Karjalainen 2009; Strid et al. 2010). Data from neutral nuclear and mitochondrial genetic markers have revealed that north European populations in Scandinavia and on Baltic islands that are isolated from the species continuous area of distribution have been founded mainly by coastal populations from Baltic States and Poland. This colonization distance is far beyond the species natural dispersal capacity. Inferred colonization pathways including good estimates for timing of establishments (literature and databases) suggest that most of these populations originate from human-mediated introductions (e.g., cargo transport) rather than natural dispersal (Kaňuch et al. 2013). Although the potential impact of introduced *M. roeselii* on the native insect communities or ecosystem functions is largely unknown (cf., Berggren and Low 2004), these populations constitute a useful system in which basic processes of human-mediated colonization and/or invasions can be studied (e.g., Allendorf and Lundquist 2003; Dlugosch and Parker 2008).

The aim of this study was to learn about the temporal dynamics of individual population genetic profiles after population establishment and to determine how genetic diversity changes in colonized but isolated populations with a known colonization history. To do this, we specifically (1) compared the level of variation in nuclear and mitochondrial markers as a proxy for overall variation in the introduced populations with the levels in the founder populations and (2) analyzed the temporal changes of genetic variation in the introduced populations. Our expectations were that the isolated populations would have a reduced genetic diversity, that the youngest populations would exhibit signs of previous bottlenecks, and finally, that the level of diversity would be related to the age of colonized populations. We expected that the patterns found can serve as an indicator of what mechanism(s) is involved. These data provide a unique and useful base for continued analyses and testing of the suggested processes.

## Material and Methods

### The study species

**Figure 1:**
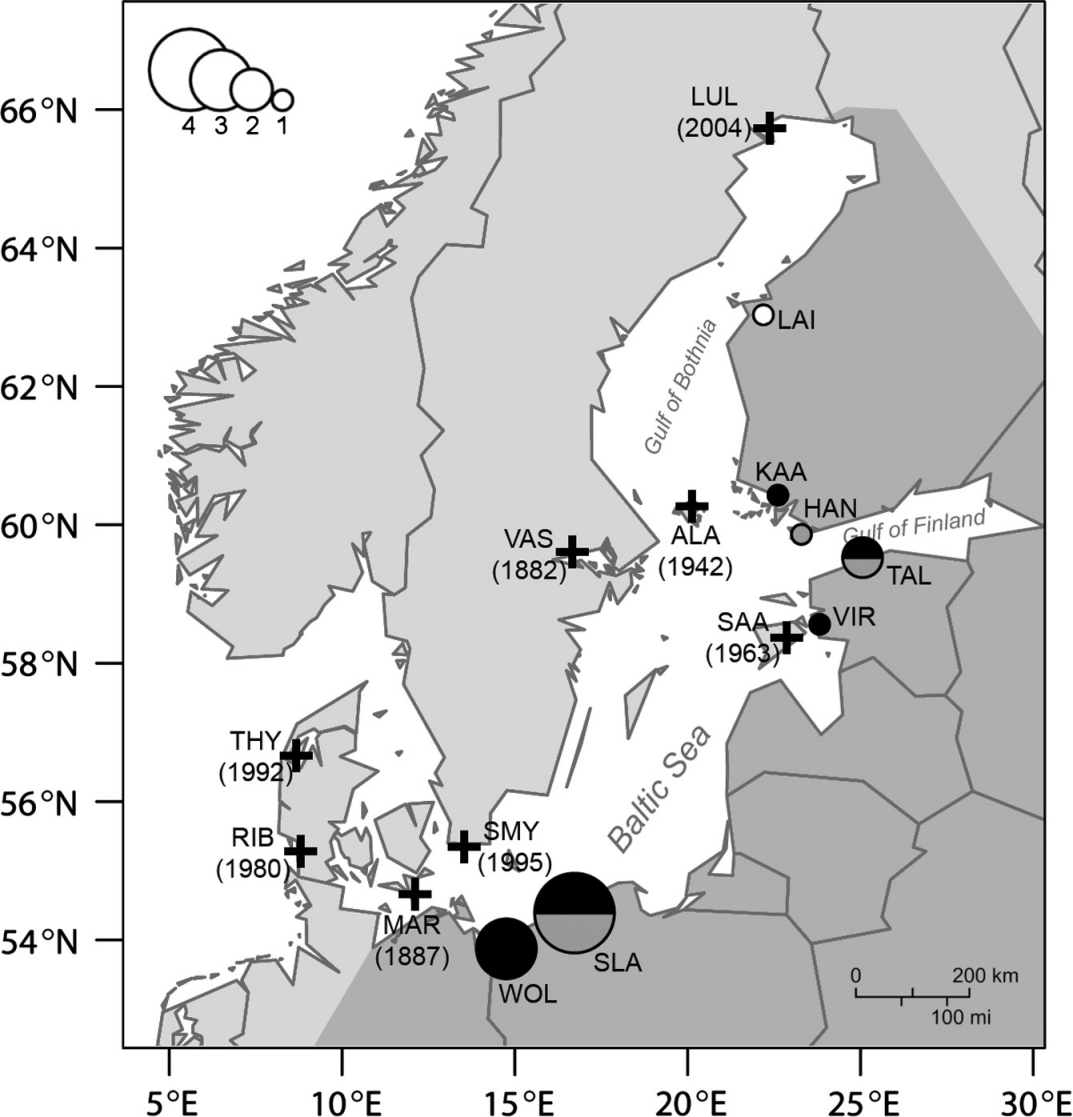
Eight isolated populations of *Metrioptera roeselii* (crosses in the light gray area on the map) and seven sites sampled in the area where the species occurs as a continuous population in northern Europe (circles in the dark gray area). The set of sites from the continuous area that is used in the analyses (Fig. 2) is a subset of the total number of populations that were sampled (see Kaňuch et al. 2013, for more details). The selection was based on the results of an assignment test, which inferred the most likely founder sources. The size of the circle in the continuous area corresponds to the number of isolated populations which were assigned to this founder population (1–4 populations). The proportion of assignment ranks is indicated by the color of the circle (black – first, gray – second, white – third rank; for details see Kaňuch et al. 2013). The year of the first published record or unpublished observation of the species in the area is shown for each isolated population.

This insect is a small (body length of 14–18 mm) and common omnivorous bush-cricket species that inhabits a wide range of grassland habitats (Ingrisch and Köhler 1998). Its natural distribution extends across mid and northern Asia to Europe, whereas in Europe, it is mainly distributed in the central and eastern parts of the continent (comprehensive data can be obtained from Eades et al. 2013). However, the species range has expanded west-and north-ward in recent years in spite of its limited dispersal ability (Fig. 1; for details, see Kaňuch et al. 2013). It has mainly one generation per year, and in northern Europe individuals reach maturity in summer (July). The males' characteristic song, that can be heard from July until the end of autumn, makes the species easy to detect and facilitates surveys and specimen collection. The females lay their eggs in hollow grass stems or other plant substrates. The material (often as hay for cattle or horses) has previously been found to work as vectors for unintentional transports of species to new sites (e.g., Wagner 2004). The nymphs hatch in spring, the first or second year after the eggs are laid, and they go through six instars before they are fully developed (Ingrisch and Köhler 1998).

### Sampling of data

Between August and September 2008, we sampled eight spatially isolated populations in Sweden, Denmark, and the Baltic Sea islands of Åland and Saaremaa where current gene flow between them and their founders is not expected (Kaňuch et al. 2013). These populations were all separated from the species' continuous range by at least 100 km of land or 50 km of sea (Fig. 1). During the same period, we also sampled individuals along the Baltic Sea coasts, predominantly from locations close to larger harbors. We sampled in Finland, Estonia, Latvia, Lithuania, Poland, and Germany where the species is abundant and occurs in a continuous population. The areas are where the isolated populations originate from according to recent phylogeographic inferences (Kaňuch et al. 2013). To enable an unbiased comparison of genetic diversity between the isolated and continuous populations, using the most likely origin of isolated ones, we selected seven of the 23 sampled sites within the species continuous distribution area (Fig. 1 and Table 1). The choice of sites was based on results from an assignment test in GeneClass 2.0 software (Piry et al. 2004). The allele frequency-based assignment of the isolated populations was in agreement with the Bayesian inferences of both microsatellite and mitochondrial markers; we were therefore confident that the selection was robust and relevant (for details, see Kaňuch et al. 2013). Overall genetic differentiation between groups of continuous and isolated populations was significant, suggesting restricted gene flow (microsatellite data, *F*_ST_ =0.025, *P *<* *0.05 after Bonferroni correction; mtDNA data, *F*_ST_ = 0.125, *P *<* *0.001). We genotyped 24 individuals in every isolated and continuous population (except in population LUL where only 17 individuals were found at the time of sampling) for seven nuclear microsatellites (239 males and 138 females in total; Table 1). We also sequenced a 676-bp fragment of the mitochondrial cytochrome oxidase subunit I (COI) gene from a maximum of four randomly chosen males and females (6–8 individuals) per population (62 males and 54 females in total; Table 1). Detailed information about genetic markers, laboratory procedures, and protocols is described in Kaňuch et al. (2013).

**Table 1:** Genetic diversity in fifteen *Metrioptera roeselii* populations in northern Europe based on seven microsatellite loci and a 676–bp-long fragment of the COI mtDNA gene. The continuous populations are considered to be founders of the isolated populations according to the results of an assignment test (details and geographic positions are presented in Kaňuch et al. 2013)

Site	*N*_msats_	*N*_COI_	Ar (min–max)	*H*_E_ (±SE)	*F*_IS_ (±SE)	Ne (±95% CI)	hd (±SD)	*π* (±SD)
Continuous populations								
LAI	14 + 10	4 + 4	7.3 (3.0–11.2)	0.760 (0.023)	0.167 (0.020)	50 (26–139)	0.857 (0.108)	0.010 (0.003)
KAA	13 + 11	4 + 4	6.5 (3.0–11.5)	0.701 (0.038)	0.222 (0.031)	24 (13–48)	0.643 (0.184)	0.005 (0.003)
HAN	19 + 5	4 + 4	6.7 (2.0–12.5)	0.671 (0.051)	0.215 (0.028)	50 (26–154)	0.893 (0.086)	0.012 (0.002)
TAL	23 + 1	7 + 1	8.9 (2.0–13.8)	0.744 (0.048)	0.208 (0.036)	46 (22–142)	0.893 (0.086)	0.009 (0.002)
VIR	17 + 7	4 + 4	9.4 (2.0–15.4)	0.738 (0.050)	0.065 (0.013)	69 (35–302)	1.000 (0.063)	0.010 (0.002)
SLA	15 + 9	4 + 4	10.3 (2.6–15.4)	0.776 (0.048)	0.190 (0.024)	69 (34–278)	0.929 (0.084)	0.010 (0.002)
WOL	18 + 6	4 + 4	9.7 (2.0–14.8)	0.762 (0.046)	0.172 (0.015)	69 (37–223)	0.857 (0.108)	0.007 (0.001)
Isolated populations								
LUL	2 + 15	2 + 6	2.8 (1.9–4.0)	0.419 (0.060)	0.078 (0.029)	15 (7–42)	0.000 (0.000)	0.000 (0.000)
ALA	12 + 12	4 + 4	6.3 (2.0–8.8)	0.711 (0.024)	0.129 (0.015)	39 (22–90)	0.000 (0.000)	0.000 (0.000)
VAS	12 + 12	3 + 3	5.7 (3.6–9.3)	0.643 (0.033)	0.093 (0.019)	26 (14–56)	0.000 (0.000)	0.000 (0.000)
SAA	23 + 1	6 + 1	6.3 (2.0–10.6)	0.676 (0.036)	0.234 (0.025)	37 (19–86)	0.810 (0.130)	0.009 (0.002)
THY	21 + 3	5 + 3	4.9 (2.9–8.8)	0.673 (0.025)	0.111 (0.021)	29 (16–63)	0.750 (0.096)	0.002 (0.000)
SMY	14 + 10	4 + 4	4.2 (1.0–8.1)	0.529 (0.059)	0.347 (0.041)	28 (14–72)	0.250 (0.180)	0.003 (0.002)
RIB	14 + 10	3 + 4	5.7 (2.0–12.8)	0.688 (0.029)	0.108 (0.016)	39 (21–95)	0.667 (0.160)	0.003 (0.001)
MAR	17 + 7	4 + 4	6.6 (2.9–9.5)	0.742 (0.030)	0.158 (0.021)	46 (23–124)	0.000 (0.000)	0.000 (0.000)

*N*_msats_, number of individuals (males + females) genotyped for seven microsatellite loci; *N*_COI_, number of individuals (males + females) sequenced for the COI gene sequences; Ar, allelic richness, that is, the mean number of alleles per locus and population, rarefied to sample size of 15 diploid individuals; *H*_E_, mean unbiased expected heterozygosity; *F*_IS_, mean inbreeding coefficient when null alleles are accounted for; Ne, effective size of population; hd, haplotype diversity; *π*, nucleotide diversity (per site).

### Genetic diversity in isolated versus continuous populations

To measure and compare genetic diversity among sites, we used six different indices. Four were based on microsatellite data and two were based on mitochondrial sequences. All microsatellite loci were tested for the presence of null alleles, effects of stuttering, and large allele dropout using MicroChecker 2.2.3 (van Oosterhout et al. 2004). As the Fisher's combined probability test found in all loci the presence of null alleles (1–15% per locus), according to suggestion by Chapuis and Estoup (2007), we calculated only allelic richness (Ar) as the mean number of alleles per locus and population, rarefied to 15 diploid individuals, and unbiased expected heterozygosity (*H*_E_) averaged over loci in the software HP-Rare 1.1 (Kalinowski 2005) and Genetix 4.05 (Belkhir K et al. 2004), respectively. When estimating the inbreeding coefficient (*F*_IS_), we used the approach of individual inbreeding model (IIM) in the software INEst (Chybicki and Burczyk 2009) which takes into account that both null alleles and inbreeding can produce an excess of homozygotes. To estimate the effective size of each population (Ne), we used a single-sample approach of the sibship method, which assumed random mating and a full likelihood model, as described by Wang (2009) and implemented in the software Colony 2 (Jones and Wang 2010). Finally, haplotype diversity (hd) and nucleotide diversity (*π*) were calculated from the mitochondrial COI sequences for each population using the software DnaSP 5 (Librado and Rozas 2009). Differences in levels of genetic variation between the two groups of populations were tested with a Mann–Whitney *U*-test applied on the values averaged across loci.

### Tests of mutation-drift equilibrium

To test for bottleneck effects in the isolated populations, if a population was affected by a recent reduction in effective size, we firstly performed Sign and Wilcoxon tests with the null hypothesis being the presence of a mutation-drift equilibrium under the two-phased mutation model (TMM). This was carried out in the software Bottleneck 1.2.02 (Cornuet and Luikart 1996). The selected TMM model should have the best fit for most microsatellite data as it combines the stepwise mutation model and the infinite allele model (Piry et al. 1999). Using the suggestions of Garza and Williamson (2001), we set up program parameters to run with 90% of single-step mutations, 10% of variance among multiple steps, and 10,000 simulation replicates. Secondly, we controlled for signatures of a bottleneck by the M-ratio method using the software MPVal and CriticalM (Garza and Williamson 2001). The total number of alleles (*k*) divided by overall range in allele size (*R*) produces ratio (M), which is expected to be smaller in recently reduced populations than in populations in mutation-drift equilibrium as it is supposed that *k* decreases faster than *R* when the population size is reduced (Garza and Williamson 2001). We simulated genetic diversity in a population with constant size at a microsatellite locus evolving under a single-step mutation model, with a mean size of multistep mutations Δ_g_ = 3.5 and the proportion of single-step mutations *p*_*s*_ = 0.89 (as suggested by Garza and Williamson 2001), and compared empirical *M* values to 95% critical values (*M*_c_) derived from 10,000 such simulations. Ancestral theta (*θ*) was set at three different values (0.1, 1, 10) to account for a wide range of mutation rates and possible Ne values prior to population bottleneck.

### Temporal change in genetic diversity

The relationship between the time since initial establishment (the age of population) and the genetic diversity of isolated populations was tested by single linear regressions for all six above-mentioned indices (Ar, *H*_E_, *F*_IS_, Ne, hd, and *π*). The estimate of a population's minimum age was based on the earliest date of observations from published reports and databases (Albrecht 1963; de Jong and Kindvall 1991; Ahlén 1995; Bavnhøj 1996; Karjalainen 2009; http://www.artportalen.se; Fig. 1). Unless noted otherwise, analyses were performed in R 2.14.1 statistical environment (R Development Core Team 2011).

## Results

**Figure 2:**
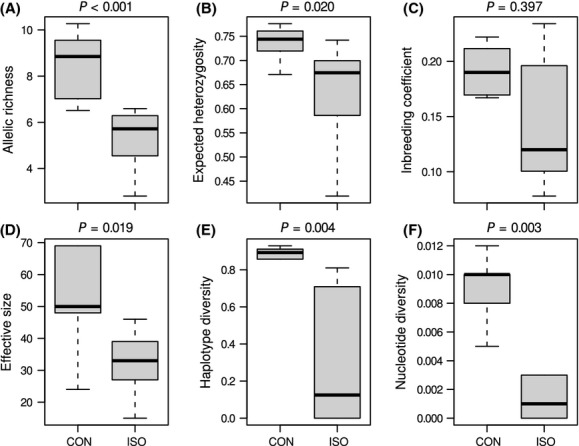
Genetic diversity in putative source populations from the continuous area of the species distribution (CON) compared with populations which are isolated from the continuous area (ISO) in northern Europe. Indices (A–D) are based on seven microsatellite markers and (E–F) on 676–bp-long fragment of the COI mtDNA gene. Box plots represent medians, 25–75% percentiles and nonoutlier ranges; *P*-values correspond to nonparametric Mann–Whitney *U*-tests.

**Figure 3:**
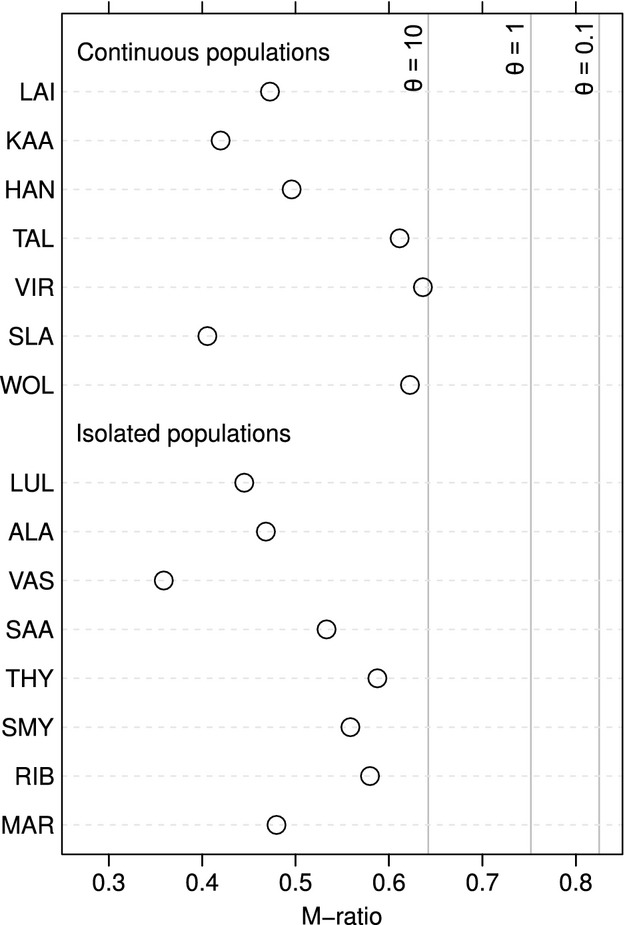
The M-ratio values for continuous and isolated populations. Based on simulations, all values are significant and indicate a bottleneck as they occur below critical *M*_c_ (vertical lines) simulated for three levels of ancestral theta (*θ *= 0.1, 1 and 10).

All genetic indices, except the inbreeding coefficient, had significantly higher values in sites within the area where the species is abundant and occurs as a continuous population (Mann–Whitney *U*-test; *P *<* *0.05; Fig. 2 and Table 1). We found reduced genetic diversity in the isolated populations of *M. roeselii* also when the isolated populations were compared with any other set of possible founders populations which were sampled along the Baltic Sea cost of the adjacent area of the continuous species range (raw data are presented in Kaňuch et al. 2013). The isolated populations had lower diversity, but different analytic approaches gave different information on the existence of a recent genetic bottleneck. A two-phased mutation model used on the data did not show signs of a bottleneck as testing across loci in the isolated and continuous populations showed no heterozygosity excess (sign test, *P *>* *0.05; Wilcoxon test, one-tailed *P *>* *0.05). Contrary, when M-ratio values were compared, they were below the M_c_ for all three levels of ancestral *θ* (Fig. 3), which indicated a recent bottleneck in all of the studied populations. There was, however, no difference in M-ratio between isolated and continuous populations (Mann–Whitney *U*-test; *P *=* *0.613). We can therefore not conclude if isolated populations are being in mutation-drift equilibrium or not.

**Figure 4:**
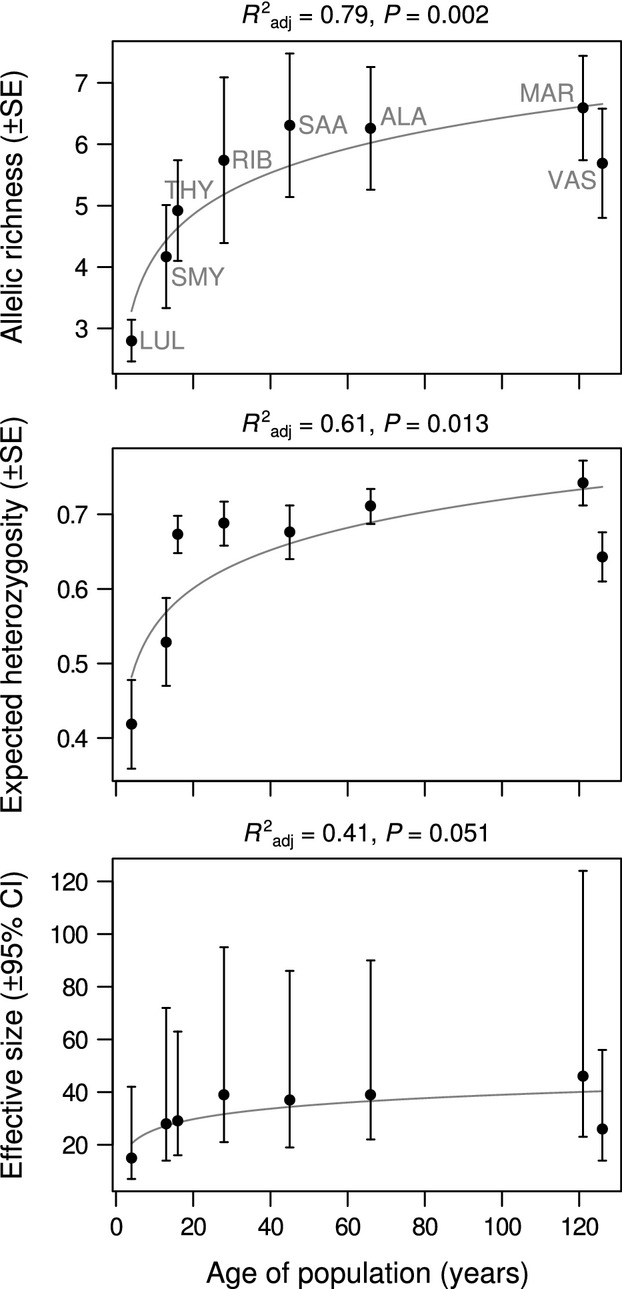
Allelic richness, unbiased expected heterozygosity, and effective size of isolated populations plotted against years since first observation or the first published record of the population. The data are based on seven microsatellite markers and fitted with logarithmic relationship in simple linear regression models.

Allelic richness, unbiased expected heterozygosity, and the effective size of the population showed a significant (Ar, *P *=* *0.002; *H*_E_, *P *=* *0.013) or close to significant (Ne, *P *=* *0.051) correlation with the age of the isolated population (Fig. 4). This regression pattern had the best fit in a logarithmic model [Ar = 1.92 + 0.98 × ln(age); *H*_E_ =0.38 + 0.07 × ln(age); Ne = 12.39 + 5.77 × ln(age)]. The youngest of the isolated populations (<20 years) showed the lowest values of genetic diversity, with diversity increasing rapidly in populations of intermediate ages and leveling off toward the oldest populations (>120 years) (Fig. 4). The other tested indices of diversity (i.e., inbreeding coefficient, haplotype, and nucleotide diversities) did not show a correlation between their values and the age of population.

## Discussion

In a previous study, Kaňuch et al. (2013) suggest that populations of *M. roeselii* established far beyond the species main distribution were most likely introduced via long-distance passive transport of individuals and/or eggs, probably with hay or other plant material from cargo ships. In the present study, we show that these events have resulted in significantly reduced genetic diversity in the introduced populations compared with their inferred sources. This is frequently observed when isolated populations lack gene flow (e.g., Lande 1988; Lee 2002; Dlugosch and Parker 2008; Simberloff 2009; Demandt 2010). More interestingly, we found a temporal pattern rarely observed in natural populations; the level of genetic diversity increased with an increasing number of generations with no sign of losses from genetic drift (Fig. 4). The regained genetic variation in the isolated populations of *M. roeselii* may be caused by different mechanisms, which can act solely or in combination.

The observed rapid increase in allelic richness and heterozygosity a short time after colonization is very similar to the pattern of gradually increasing diversity that Le Corre and Kremer (1998) modeled in response to an increasing number of colonists and migrants. As multiple introductions are important for the colonization success (Roman 2006; Facon et al. 2008), possible reoccurrences of passive transports could enhance demographic growth and genetic diversity in our established isolated populations (Pannell and Charlesworth 2000; Allendorf and Lundquist 2003). Although the species' genetic structure, inferred by microsatellites and the geographic distribution of mtDNA haplotypes, suggests that introductions most likely originate from a restricted region within the continuous area (Kaňuch et al. 2013), recurrent introductions and hybridization between different founding populations are still possible if trading and transport are or have been extensive. Moreover, this would be the most realistic mechanism increasing genetic variation in such isolated populations (Facon et al. 2008; Demandt 2010).

However, empirical data on temporal changes of genetic diversity in isolated populations established from multiple introductions suggest a different, *U*-shaped pattern (Dlugosch and Parker 2008). In order to make a useful comparison with our pattern, we would need to compare our data with only the microsatellite data from Dlugosch and Parker (2008) meta-analysis. Further, another limitation for a comparison is that the author's data were not represented for the entire time scale; the first 50 years after introduction are missing from their study, and this is the period where we observed the most rapid change (Fig. 4). When considering these limitations, the same type of regression analysis as Dlugosch and Parker (2008) supports a logarithmic relationship rather than a quadratic one in both allelic richness (*R*^2^ = 0.79) and expected heterozygosity (*R*^2^ = 0.61) on the time scale from 4 to 126 years. One could argue that our logarithmic relationship is strongly influenced by the low genetic diversity of LUL at the northern periphery and that this population inhabits an unrepresentatively unfavorable climatic cold area which resulted in very low effective size. However, rapid current expansion at the northern species range within the continuous area (Karjalainen 2009) does not suggest that climatic conditions have negative effects on the populations' performance. Finally, another difference that may explain the lack of similarity between our study and Dlugosh and Parker's is that the data are compiled from studies on different organisms. The complexity of colonization processes differs in plants, fungi, and animals, due to the different mechanisms of dispersal, and this is partly why it is difficult to find universal patterns of temporal change in genetic diversity of isolated populations (cf. Le Corre and Kremer 1998).

Although we are missing empirical data on this, we suggest also another possible mechanism causing the rapid increase in genetic variation in the isolated populations of *M. roeselii* – an exceptional rapid intrapopulation mutation rate. Indirect evidence of rapid mutation rates in neutral loci, based on higher proportion of long microsatellites (relative to other orders of insects), has previously been observed in Orthoptera, although the mechanism behind this is generally unknown (Chapuis et al. 2012). A fast rate of molecular evolution is plausible in species with relatively quick generation turnover, high fecundity, and short lifespan (Bromham 2011). If we roughly calculate the mutation rate required to create an average of three new mutations per microsatellite locus during the first 30 years (this is equal to the number of generations) after establishment, with effective population size of 40 individuals (Fig. 4), we get a mutation rate per nucleotide site of about 10^−5^ bp per generation. As it seems that mutations can occur more frequently in marginal or isolated populations (Agrawal and Wang 2008; Bromham 2011), we suggest that this mechanism may contribute to the pattern we found in this study. However, we are fully aware of that this needs to be carefully tested as the calculated value of theoretical mutation rate in *M. roeselii* is beyond the upper limit of expected average for invertebrates (Lynch 2010).

Association between genetic diversity and population dynamics is considered fundamental for species survival (Allendorf and Lundquist 2003). The fact that we found no evidence of that the populations suffer from recent bottlenecks and high inbreeding level (Figs 2 and 3), and that the youngest isolated populations had similar levels of *F*_IS_ as the old populations (Table 1, Fig. 1), makes us believe that the suggested mechanisms are the reasons why *M. roeselii* so successfully currently colonizes northern Europe. The species appears to be highly adaptable (Cassel-Lundhagen et al. 2011), and it is rapidly expanding its distribution range in our study area (Preuss 2012). Hence, we suggest that small-sized and isolated populations of *M. roeselii* are probably not subjected to a loss of fitness (cf. Falconer and Mackay 1996; Hedrick and Kalinowski 2000; Keller and Waller 2002; Ortego et al. 2011). Populations that are capable of avoiding detrimental effects caused by a restricted gene flow can support the previous findings from large-scale introduction experiments where *M. roeselii* successfully thrived and colonized uninhabited areas even when founded by very small propagule sizes (Berggren 2001).

## Data Accessibility

Detailed data for microsatellite markers and GenBank accession numbers to DNA sequences used in this study are provided in the paper by Kaňuch et al. (2013).
